# Medium and long-term adherence to postabortion contraception among women having experienced unsafe abortion in Dar es Salaam, Tanzania

**DOI:** 10.1186/1471-2393-8-32

**Published:** 2008-07-31

**Authors:** Vibeke Rasch, Fortunata Yambesi, Siriel Massawe

**Affiliations:** 1 Department of International Health, Immunology and Microbiology, University of Copenhagen, Denmark; 2 Temeke Municipal Hospital, Dar es Salaam, Tanzania; 3 Muhimbili Medical Centre, Dar es Salaam, Tanzania; 4 Division of International Health (IHCAR), Department of Public Health Sciences, Karolinska Institutet, SE-171 76, Stockholm, Sweden

## Abstract

**Background:**

Postabortion contraceptive service is considered an effective means in addressing the problem of unsafe abortion; in spite this fact this component remains one of the weakest parts of postabortion care. In this context, the paper aims to describe the impact of a postabortion contraceptive service intervention among women admitted with complications from unsafe abortions and to explore the women's long-term contraceptive adherence.

**Methods:**

392 women having experienced unsafe abortion were identified by an empathetic approach and offered postabortion contraceptive service, which included counselling on HIV and condom use. Questionnaire interviews about contraceptive use were conducted at the time of inclusion and 12 months after the abortion. Additionally, in-depth interviews were performed 6–12 months after the abortion.

**Results:**

Eighty-nine percent of the women accepted postabortion contraception. Follow-up information was obtained 12 months after the abortion among 59 percent of the women. Among these, 79 percent of the married women and 84 percent of the single women stated they were using contraception at 12 months. Condom use among the single women increased significantly during the 12 months follow up.

**Conclusion:**

Postabortion contraceptive services appear to be well accepted by women who are admitted with complications after an unsafe abortion and should thus be recognized as an important means in addressing the problem of unsafe abortion. In addition, counselling about HIV and condom use should be considered an essential aspect of postabortion care.

## Background

Maternal health is one of the main global health challenges and reducing the maternal mortality ratio by three-quarters by 2015 is the target for the fifth Millennium Development Goal. However, this goal is the one towards which the least progress has been made [1]. Of all maternal deaths, those related to unsafe abortions are the most severely underestimated and yet at the same time also the most easily preventable. The case fatality rate associated with unsafe abortion is estimated to be 367 deaths per 100,000 unsafe abortions, which is hundreds of times higher than that for safe and legal abortions in developed nations [2].

In spite of the compelling logic of preventing future unplanned pregnancies by providing postabortion contraceptive services to women admitted with abortion complications, this component remains one of the weakest parts of postabortion care [3,4]. This irrespective of important evidence from Burkina Faso [5], El Salvador [6], Kenya [7], Mexico [8-10], Peru [11], Russia [12], and Senegal [13] demonstrating that it is possible to increase contraceptive acceptance among women who have experienced abortion. One of the concerns in postabortion contraceptive service delivery is women's medium- and long-term adherence. A Zimbabwean study has revealed that women who received ward-based postabortion contraceptive services had higher contraceptive use for at least one year after hospital discharge, as well as fewer unplanned pregnancies and abortions [14]. In addition, it has been documented that postabortion contraceptive service delivery involving training in counseling skills increases contraceptive use up to 12 months after abortion [12]. The two mentioned studies did, however, not make any distinctions between women having experienced an unsafe abortion and women having experienced a spontaneous abortion. This in spite of the fact that these two groups of women may be expected to have quite different reproductive priorities and concerns which may affect their contraceptive acceptance as well as their contraceptive adherence.

To better understand the acceptance of postabortion contraceptive services, the present study focuses on women having experienced an unsafely induced abortion and describes these women's long-term contraceptive adherence and their experienced barriers.

## Methods

### The quantitative study

**Figure 1:**
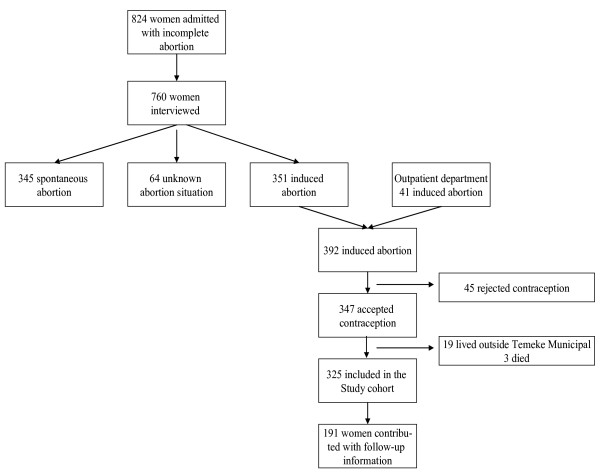
Study population, Women attending Temeke Municipal Hospital with unsafe abortions.

The study was conducted in Tanzania, which is a country where abortion is only legally available if the pregnancy is a threat to the woman's life [15]. Data were collected among women who attended Temeke Municipal Hospital (TMH) in Dar es Salaam with an abortion-related diagnosis in the period July 2001 to July 2002. The study population is presented in Figure 1, 824 women were diagnosed as having abortion complications and 760 of these women were approached and interviewed by using the previously described empathetic approach [16]. According to this approach, a woman is classified as having had an unsafe abortion if she, through an empathic dialogue, reveals that she has had an unwanted pregnancy, which she had terminated by an abortion. In all 392 women (52 percent) were classified as having had unsafe abortions and 347 of these women accepted contraceptive services and were thus included in the study.

#### Contraceptive adherence

The women were counselled in detail about contraception and how to avoid becoming infected with HIV and thereafter offered ward-based contraceptive service. Follow-up data were obtained 12 months after the women had had the abortion by a combination of hospital-based and home-based interviews [17]. In relation to the 12 months' interviews detailed information about the women's present contraceptive use, their possible motives for not using contraception, and pregnancies experienced during the observation period was obtained.

#### Follow-up

The women eligible for follow-up comprised 325 women, 191 of these women (59 percent) contributed with follow-up information; 90 returned spontaneously to the hospital for follow-up, whereas 101 were visited at home and interviewed. Among the 134 women who were lost to follow-up, 38 refused to be visited at home. Attempts were made to make home visits to the remaining 96 women among whom 32 had provided an unspecific address, 64 had, according to information from their neighbours or relatives, moved or were away from home for a longer period due to either work or family obligations.

#### Statistics

Data were entered twice by two different operators using the software Epi Info version 6.04 from Epidemiology and Disease surveillance from the CDC, Atlanta, USA. The two sets were compared and the questionable entries were reconciled. Statistical analyses were carried out by the Statistical Package for the Social Sciences (SPSS, version 12.0).

### The qualitative study

The qualitative part of the study was designed to shed light on the women's concerns related to contraceptive use, their possible motives for not using contraception, their perceived HIV risk and their motives for using or not using condoms.

In-depth interviews were carried out 6–12 months after discharge with 10 married women and 10 single women. A thematically structured interview guideline with open-ended questions was developed. The interviewer used the guideline questions to focus the discussion, but was encouraged to probe respondents whenever it was found relevant. The interviews took 30–40 minutes and were carried out in Kiswahili. All interviews were tape recorded, transcribed and later translated into English.

The strategy used to classify and evaluate the data was predetermined by the guideline; e.g. contraceptive use, motives for not using contraception, perceived HIV risk and condom use. From the transcribed conversations these themes were listed together and all data that related to the classified themes were identified. In the next step of the analyses the identified themes were combined and catalogued according to the women's marital situation.

### Ethics

All the women were informed that participation in the study was voluntary and that it would have no consequences for their further treatment whether they participated or not. Informed consent was obtained. All women who participated in the study were asked whether they would accept to be visited at home and only women who accepted this were attempted visited. Further, in relation to the home visits the women's abortion procedure was kept strictly confidential, the interviews only focused on the women's contraceptive use and no references were made to the fact that they had had an unsafe abortion. The study was ethically approved by the National Institute of Medical Research in Tanzania and by the Scientific-Ethical Committee at Karolinska Institute in Sweden.

## Results

### The quantitative study

The demographic characteristics of the cohort are presented in table 1. The majority (55 percent) of the women were below 25 years and many were single (50 percent). Women aged 19 years or below were more likely to be lost to follow-up. Likewise, less follow-up information was obtained among single women and women who had not given birth previously.

**Table 1:** Demographic characteristics among women who had unsafe abortions and follow-up information achieved at 12 months

	Study cohort		Follow-up information		
	
	N = 325	%	Achieved N = 191 %	Not achieved N = 134 %	P value
Age					
-19 years	70	22	18	27	0.09
20–24 years	108	33	32	35	
25–30 years	79	24	26	22	
30+ years	68	21	25	16	
Marital situation					
Married	161	50	58	38	0.003
Single	145	45	38	54	
Divorced/wid.	18	6	4	8	
Education					
≤ Std 4	46	14	12	17	0.305
Std 5–7	239	74	77	69	
≥ Form 1	39	12	11	13	
Previous births					
0	108	33	2	6	0.003
1	73	23	34	45	
2	61	19	27	27	
3+	82	25	36	22	

#### Contraceptive acceptance

The 392 women who had experienced an unsafe abortion were offered contraceptive counselling and service, 347 (89 percent) accepted the service offered and left the ward with a contraceptive method according to their own choice.

#### Contraceptive use before and after the abortion

Contraceptive use before the abortion and 12 months after the abortion was assessed among women who accepted postabortion contraceptive services (table 2). Before the abortion, 18 percent of the married women had ever used hormonal contraception, that be oral contraception or medroxyprogesteron injections, the same applied for 14 percent of the single women. Twelve months after the abortion, the proportion of women who stated they were using oral contraception or medroxyprogesteron injections had increased significantly among both married and single women. Hence, 77 percent of the married women and 84 percent of the single women stated they were using hormonal contraception when interviewed 12 months after the abortion

**Table 2:** Contraceptive practice before and after the abortion among married and single women

	Married					Single				
	
	Pre Abortion N = 110 %		Post abortion* N = 110 %		P value	Pre Abortion N = 81 %		Post abortion* N = 81 %		P value
Condom	12	11	11	10	NS	26	32	23	28	NS
Oral contracep	14	13	45	41	< 0.000	9	11	38	48	< 0.000
Medroxyprogesteron inj.	5	5	39	36	< 0.000	3	3	29	36	< 0.000
No use	79	72	23	21	< 0.000	43	53	13	16	< 0.000

* Adds more than 100% since some women used both condom and hormonal contraception

#### Condom use

In relation to the postabortion contraceptive service offered, it was stressed that condom use also was a means to avoid STIs/HIV infections. As indicated in table 3, single women were more likely to follow this advice. Hence among the single women, a significantly higher proportion stated they had used condoms more than half of the times they had had intercourse when interviewed 12 months after the abortion in comparison with before the abortion, whereas no significant difference in condom use was found among married women.

**Table 3:** Condom use last month among married and single women

	Married					Single				
	
	Pre Abortion N = 110 %		Post abortion* N = 110 %		P value	Pre Abortion N = 81 %		Post abortion* N = 81 %		P value
> 1/2 the times	4	4	8	7	NS	8	10	18	23	<0.05
1/2 the times	3	3	2	2	NS	12	15	3	4	<0.05
< 1/2 the times	103	94	99	91	NS	61	75	59	74	NS

*Information about condom use were missing among 1 married and 1 single women

### The qualitative study

To get more detailed information about the women's priorities and concerns in relation to their contraceptive use a number of in-depth interviews were performed among single and married women 6–12 months after their abortion. The findings from the in-depth interviews were in accordance with the survey results; hence the majority of the women had been using contraception after they had been discharged from the hospital. The in-depths interviews did, however, also reveal that many women for various reasons found it difficult to find the ideal method and found it difficult to use the method consistently.

#### Barriers in relation to contraceptive use among married women

Focusing on the married women, they were often concerned about their economical situation and how expensive it was to raise a child. Many felt they already had the number of children they were able to take care of and therefore used contraception to avoid a repeated unwanted pregnancy. However, they were often caught between a wish to limit their family size and fear of real or imagined side effects and were trying to navigate between these two conflicting concerns. As Theresa (46 years) explained:

Life is very hard nowadays so it is very important to join the family planning program. I have previously tried other methods, once I used the pills and suffered from loss of appetite. Then I had the IUD inserted and suffered from severe abdominal pain so I had it removed ......Immediately after the abortion I was given the injection and I am now on my second injection.

Another concern in relation to contraceptive use among married women was the problem of transportation fee; the women did, therefore, not always return in time for new supplies and were instead relying on less effective methods such as abstinence or withdrawal. This situation is illustrated by Rita (30) who had six children:

I have come a bit late as we have transport problems, especially now with the rain. I am also living very fare from the hospital. I have to take more than one bus and the fare can come to 1000 TSH (0.86 USD). I am one month late but my husband and I have been very careful regarding this issue and we have been abstaining till now. Today when we got the fares we decided that I had to come.

A number of married women were quite determined to limit their family size and opted for highly effective contraceptive methods such as e.g. sterilization. This was the situation for Grace, a 30 year old married woman, who had seven children. When Grace was discharged from the hospital, she was offered postabortion contraceptive service. She decided to use medroxyprogesteron injection and returned to the hospital after 3 months to have the injection renewed. She did, however, not show up for the six and nine months follow-up. A home based interview was performed 12 months after the abortion and Grace explained the following about her contraceptive use:

I had financial problems [and did therefore not return to the hospital for new supplies] and right now we are not using any contraception instead my husband is practicing withdrawal. We have discussed that I latter will have to have a sterilization operation performed.

#### Barriers in relation to contraceptive use among single women

The single women had, not surprisingly, other priorities and concerns in relation to contraceptives than married women. Their main priority was to postpone childbirth until either they were married or until they had finished their education. Some of the girls were rather independent of their partner and felt they themselves had the decision making power both regarding the abortion and whether they should use a contraceptive method after the abortion. Crystle was a 16 year old single girl and her boyfriend was a couple of years older. She explained:

When I experienced I was pregnant I told my boyfriend that I wanted to have the pregnancy terminated. He was against it but I decided to go ahead. My sister in law and my boyfriend's father gave me money. My boyfriend was annoyed and he blamed his father a lot.... I am not seeing him anymore. I want to work until I am 28 then I can get married.

It was, however, not all single women who had the same knowledge and the same decision making power as Crystle. Some of the single women, who were sexually active, had no intention of becoming pregnant and did not use any contraception. These women were particularly at risk of experiencing a repeated unwanted pregnancy. For instance, Annie, who was 16 years and admitted to the hospital with complications from her second unsafe abortion, said about her contraceptive use:

After having had the first abortion I was informed about different contraceptive methods ... I decided to use the pills .... When I came home from the hospital I talked with my sister and she said that it was not yet time for me to use the pills and she took them. Both my boyfriend and my sister advised me not to use contraception. Even myself I do not want to use pills because I might not be able to become pregnant later if I use the pills.

#### Perceived HIV risk and condom use among married women

Many women explained that they found it difficult to introduce the condom in their relationship. They were particularly afraid that raising the issue would create uncertainty about whether the couple was being faithful to each other. Hence, suggesting condom use would be interpreted by the partner as his wife suspecting that he was being unfaithful and perhaps infected either with an STI or HIV. As stressed by Claire (30) who had been married to her husband for five years:

After coming back from the hospital I informed my husband about what the nurses had said and he accepted that it would be a good idea if I used the pills. I was also advised to use the condom, but I am sure my husband does not want to hear about it. If I tell him, he will ask whether I think he has a disease.

Further, many of the married women trusted their partners and saw no reason to use condoms as they did not consider themselves at risk of becoming infected with HIV. Rita was 40 years old and had six children. She used injections to avoid experiencing another unwanted pregnancy, and she explained the following:

I think the implant could be good to use. The pills are having side effects and are difficult to remember to take, whereas I have never used the condom. I see no use in using condoms as I am only sleeping with my husband. He too is not in favour of using the condoms since I am using injection. When asked about the risk of becoming infected with HIV, Rita said: I believe he has no other partner. It is good to trust each other and we do have this trust that is why we are not using condoms.

#### Perceived HIV risk and condom use among single women

The single women were generally more open-minded towards condom use, both at the time of enrolment in the study and at the 12 months follow-up. Especially the young girls seemed to be in favour of condoms; used either alone or in combination with hormonal contraception. Judith, for example, who was 20 years old, was using both condoms and oral contraceptives, but had only informed her partner about the condom:

After the abortion I was told that the pills were for prevention of pregnancy and the condoms were for prevention of sexually transmitted diseases such as syphilis and HIV. After I left the hospital I told my lover that we had to use the condoms I had got at the hospital and he agreed. He is not aware that I am also using the pill. I think it is best not to tell him as it will be of no help to him. If I tell him, he might be against me using them.

However, the majority of the single girls did not use condoms regularly and their motives for this differed. Some trusted their partners, others had introduced the condom but their partners were against using it and still others thought that the condom was mainly to be used at the beginning of a relationship, as for instance Maria (aged 20). Maria was single and had one daughter aged 4. Her relationship with the man, who was responsible for the pregnancy, had ended after the abortion. She now had a new partner, who she considered a fiancé. The importance of double protection in relation to inclusion in the study had been stressed to Maria and she had chosen to use Medroxyprogesteron injection and the condom. She was still using the injections but when asked about condom use Maria said:

My relationship with the man, who had caused the pregnancy, stopped after the abortion. I am now having another boyfriend who is my fiancé; he went to my home town and introduced himself. I used the condom the first time we were having sex but after he has introduced himself to my family I have stopped using condoms with him.

## Discussion

The vast majority of the postabortion women accepted postabortion contraceptive services and stated they were using contraception 12 months after the abortion. Follow-up information, however, was only achieved among 59 percent of the included women and the long-term contraceptive adherence among the 41 percent of the women who were lost to follow-up is unknown. The fact that abortion is illegal in the studied setting is likely to have contributed to the high drop-out rate. Further, whether illegal or not, an induced abortion is often considered a traumatic or even stigmatising event, which women would like to avoid being confronted with at a later stage in their life – a fact that is likely also to have had a negative impact on the follow-up rate. Similar problems with high drop-out rates have been experienced in other studies focusing on the long-term impact of postabortion care [14].

The high drop-out rate experienced in the present study raises the question of the extent to which the women who contributed with follow-up data differ systematically from those who did not. Loss to follow-up was more pronounced among young, unmarried women who had not given birth previously. In Tanzania, as well as in many other low income societies, premarital sex is considered immoral [18], and for fear of their abortion experience being disclosed, young or single women may thus have been more likely to provide wrong addresses to avoid a home visit, even though they accepted such a visit when they were enrolled in the study. In addition to the abortion related stigma, a high migration rate is likely also to have had a negative impact on the proportion of women who contributed with follow-up data. In Temeke Municipal, many settle in unplanned squatter areas with social problems and are likely to move when a better housing possibility becomes available, a situation which is reflected in a high yearly immigration rate of 25 percent [19]. This situation that is likely to be more pronounced among young and single women, who have not yet established themselves with their own family, may also partly explain the low follow-up rate achieved.

The findings form the present study, although hampered by a high drop-out rate, adds to the evidence that women having experienced unsafe abortions are likely to accept and use contraception. One explanation behind the high contraceptive prevalence rate found in this study may be that the service was offered as a ward based activity by well trained counsellors. An assumption which is supported by a Kenyan study where postabortion contraceptive service was accepted by 75 percent when it was on the ward, while only 41 percent obtained a method when asked to visit a separate site within the same hospital after discharge [7]. In the present study, the women were further asked to return to the hospital every 3 month to discuss their contraceptive use and have experienced problems addressed. This approach may additionally have had a positive impact on the women's contraceptive adherence. Hence, women who have experienced an induced abortion may be reluctant to use contraception due to lack of detailed knowledge about contraception and fear of experienced or imagined side effects [20,21]. Frequent counselling that addresses the women's concerns may therefore increase the women's contraceptive adherence. Whether the approach of inviting the women to return to the hospital for ongoing counselling and service is the most appropriate way to strengthen the women's ongoing contraceptive use can be discussed. Hence, at a programmatic level, the cost-effectiveness of such an approach may be questioned and it may be more relevant to refer the women to a primary health facility.

Postabortion contraceptive services are apparently well accepted by women who are admitted with complications after an unsafe abortion and should thus be considered an important means in addressing the problem of unsafe abortions. However, while hospitals incur only minor additional costs by introducing postabortion contraception, few have done so successfully. To begin with, contraceptive services and incomplete abortion treatment are typically delivered and administered separately – in different areas of one building, in separate buildings, or even within different institutions. In addition to separately administered services, lack of appropriate training, time, and institutional support can make physicians and nurses in the wards where patients receive postabortion care reluctant or unable to take on the duty of providing contraceptive services. To ensure postabortion contraceptive services are being offered in a comprehensive and sustainable way the following requirements should be addressed: qualified contraceptive counsellors should be present in the gynaecological ward; the most commonly used contraceptive methods (oral contraceptives, injections and condoms) should be made available in the ward and; the service should be monitored and evaluated through a simple routine supervision system.

High HIV prevalence rates, ranging from 19 percent to 25 percent, have been found among Tanzanian single women who have experienced an unsafe abortion [22]. These figures are considerable higher than sentinel surveillance results reporting HIV prevalence rates of 10–14 percent among pregnant women in Temeke Municipal. Therefore, in Tanzania as well as in other areas with high HIV prevalence rates, counselling about HIV and condom use should be considered an essential aspect of postabortion care. The present study documents that such counselling may lead to an increased number of single women practising safe sex. It has been documented that the vast majority of Tanzanian women are aware of risky behaviour and the protective role of abstinence, faithfulness to one uninfected partner and condom use [23]. However, as the present study shows, they face a number of obstacles in translating their knowledge into safer sex practices. Both women and men associate condoms with promiscuity, STIs and HIV. Suggesting condom use may, therefore, imply either that one has a sexually transmitted disease or that one mistrusts one's partner [24]. This association of condom use with promiscuity undermines the use of condoms as an HIV-preventive measure, especially among the married women. These difficulties related to condom use should be acknowledged when counselling married women on contraceptive use. Especially, since marriage has been found to be a significant risk factor for HIV infection in Tanzania as well as in other sub-Saharan African countries [25].

## Conclusion

Legal reforms ensuring women easy access to safe legal abortions is the primary option for addressing the tragic toll of unsafe abortion. However, this is not likely to take place in a foreseeable future in many low-income coutries; meanwhile there is a need for comprehensive postabortion care programs that ensure women prompt access to high-quality postabortion care. In that relation it should be acknowledged that postabortion women are likely to accept and use contraception when the service is offered as an integrated part of postabortion care.

## Competing interests

The authors declare that they have no competing interests.

## Authors' contributions

VR designed the study, performed the analyses, and led the writing. FY and SM assisted in designing the study and supervised the implementation of the study. All authors contributed to the interpretation of the findings and reviewed drafts of the paper.

## Pre-publication history

The pre-publication history for this paper can be accessed here:


